# *PARD3* gene variation as candidate cause of nonsyndromic cleft palate only

**DOI:** 10.1111/jcmm.17452

**Published:** 2022-07-04

**Authors:** Renjie Cui, Dingli Chen, Na Li, Ming Cai, Teng Wan, Xueqiang Zhang, Meiqin Zhang, Sichen Du, Huayuan Ou, Jianjun Jiao, Nan Jiang, Shuangxia Zhao, Huaidong Song, Xuedong Song, Duan Ma, Jin Zhang, Shouxia Li

**Affiliations:** ^1^ Key Laboratory of Metabolism and Molecular Medicine, Ministry of Education, Department of Biochemistry and Molecular Biology, Collaborative Innovation Center of Genetics and Development, Institutes of Biomedical Sciences, School of Basic Medical Sciences, Shanghai Medical College Fudan University Shanghai China; ^2^ Shanghai Ninth People’s Hospital Shanghai JiaoTong University School of Medicine Shanghai China; ^3^ Department of Clinical Laboratory Central Hospital of Handan Hebei China; ^4^ Oral and Maxillofacial Surgery Central Hospital of Handan Hebei China; ^5^ Children’s Hospital of Fudan University Shanghai China

**Keywords:** NSCP, nonsyndromic cleft palate only, orofacial clefts, PARD3

## Abstract

Nonsyndromic cleft palate only (NSCP) is a common congenital malformation worldwide. In this study, we report a three‐generation pedigree with NSCP following the autosomal‐dominant pattern. Whole‐exome sequencing and Sanger sequencing revealed that only the frameshift variant c.1012dupG [p. E338Gfs*26] in *PARD3* cosegregated with the disease. In zebrafish embryos, ethmoid plate patterning defects were observed with PARD3 ortholog disruption or expression of patient‐derived N‐terminal truncating *PARD3* (c.1012dupG), which implicated PARD3 in ethmoid plate morphogenesis. PARD3 plays vital roles in determining cellular polarity. Compared with the apical distribution of wild‐type PARD3, PARD3‐p. E338Gfs*26 mainly localized to the basal membrane in 3D‐cultured MCF‐10A epithelial cells. The interaction between PARD3‐p. E338Gfs*26 and endogenous PARD3 was identified by LC–MS/MS and validated by co‐IP. Immunofluorescence analysis showed that PARD3‐p. E338Gfs*26 substantially altered the localization of endogenous PARD3 to the basement membrane in 3D‐cultured MCF‐10A cells. Furthermore, seven variants, including one nonsense variant and six missense variants, were identified in the coding region of *PARD3* in sporadic cases with NSCP. Subsequent analysis showed that PARD3‐p. R133*, like the insertion variant of c.1012dupG, also changed the localization of endogenous full‐length PARD3 and that its expression induced abnormal ethmoid plate morphogenesis in zebrafish. Based on these data, we reveal *PARD3* gene variation as a novel candidate cause of nonsyndromic cleft palate only.

## 1 INTRODUCTION

Orofacial clefts (OFC1 [MIM 119530]) are a common craniofacial birth defect with significant medical, psychological, social and economic ramifications, affecting one in seven hundred live births worldwide.^1^ Orofacial clefts can be anatomically divided into cleft lip and palate (CLP), cleft lip (CL) and cleft palate only (CP). The distinct original mechanism of development and formation of the lip and palate suggests that CP is a developmentally distinct malformation from both CL and CLP.^2^ CP is responsible for 33% of orofacial clefts, affecting 1–25 out of 10,000 newborns throughout the world, and approximately 50% of CP cases are classified as nonsyndromic clefts.^3^, ^4^ To date, more than 26 risk loci and several candidate genes have been identified in nonsyndromic cleft lip with or without palate.^5^, ^6^, ^7^, ^8^, ^9^ In addition, recent studies have identified pathogenic variants in *ARHGAP29* in CP.^10^, ^11^ Recently, a sequencing study identified the dominant *GRHL3* mutations as an important cause of nonsyndromic cleft palate.^12^ Analyses of 258 NSCP case‐parent triads from Europe revealed that a missense variant of *LOXL3* under a recessive model was associated with NSCP.^13^ Even so, the genetic aetiology of NSCP is still largely unclear.

Cell polarity is defined as asymmetry in cell shape, protein distributions and cell functions, and most eukaryotic cells show clear and obvious polarity.^14^, ^15^ Apical–basal polarity and planar cell polarity perpendicular to the apical–basal axis are thought to play a synergistic effect in biological processes such as epithelium migration, epithelium‐specific adhesion and neural development.^14^, ^15^, ^16^, ^17^, ^18^, ^19^ The existence of cell polarity plays a crucial role in palatogenesis by instructing the formation of a variety of cell–cell junctions, including tight junctions and adherens junctions.^20^, ^21^ In E14.0 mouse embryonic development, the reduced apicobasal polarity of epithelial cells proposes a mechanism that helps to avoid the apex–apex contact of epithelial cells of apposing palatal shelves and to unify the epithelium of bilateral shelves in palatogenesis.^22^ Mutations in the interferon regulatory factor 6 (*IRF6*) gene have been identified as the causative mutation of some nonsyndromic and syndromic orofacial clefts, and a related study showed that Irf6 conditional knockout led to the disturbance of cell polarity in the development of the orofacial region histologically.^23^ Knockout of Meox‐2, a gene involved in anterior–posterior polarity establishment, resulted in cleft palate by the breakdown of newly fused palates.^24^

Par‐3 Family Cell Polarity Regulator (PARD3 [OMIM 606745]) belongs to a family of PARD proteins that are required for several biological processes, including polarized cell growth, cell division and the formation of epithelial tight junctions.^25^, ^26^, ^27^ PARD3, a scaffold protein that was first identified in *C. elegans*, contributes to the development of junctional structures and apical–basolateral polarization.^28^

In this study, we report one pedigree comprising six affected individuals with nonsyndromic cleft palate only. Whole‐exome sequencing was performed in three affected individuals, and the truncating variant located within the N‐terminal coding sequence of PARD3 was identified as the cause in the pedigree.

## 2 MATERIALS AND METHODS

### 2.1 Clinical samples

The 3‐generation 18‐member family (pedigree) with NSCP and unrelated controls were recruited and enrolled by the Central Hospital of Handan, Hebei, China. The 57 sporadic NSCP cases were recruited and enrolled by Children’s Hospital of Fudan University and Shanghai Ninth People’s Hospital affiliated with Shanghai Jiao Tong University School of Medicine. All the studies were approved by the ethics committee of the Central Hospital of Handan City. All procedures were carried out only after written informed consent had been obtained from all research participants and from the parents of members younger than 18 years of age, as stipulated by the Declaration of Helsinki. Peripheral blood from all research participants was obtained, and genomic DNA was extracted according to standard protocols.

### 2.2 Exome sequencing and validation

Whole‐exome sequencing (WES) was performed to identify candidate pathogenic gene variants in individuals from the pedigree. Genomic DNA was extracted from whole blood samples using a blood DNA kit (Qiagen), and 1 μg of purified gDNA was fragmented. Exome libraries were prepared using the TruSeq DNA LT Sample Prep Kit and TruSeq Exome Enrichment Kit (Illumina, Inc.) according to the manufacturer's protocols. Sequencing was conducted on an HiSeq 2500 (Illumina) to generate paired‐end reads. The raw sequences that met quality standards were aligned to b37/hg19 using Burrows–Wheeler Aligner software (BWA).^29^ Mutational screening of *PARD3* was then performed by targeted sequencing and Sanger sequencing in 57 sporadic NSCP samples.

### 2.3 Zebrafish breeding

The AB zebrafish line (ZFIN ID: ZDB‐GENO‐960809‐7) was used in this research. All wild‐type and genetically modified zebrafishes were bred and maintained at the Fudan University Zebrafish Breeding Centre. Embryos were grown in egg water (0.3 g/L NaCl, 75 mg/L CaSO_4_, 37.5 mg/L NaHCO_3_ and 0.0003% methylene blue) and maintained at 28.5°C. All zebrafish experimental procedures were conducted in accordance with the Institutional Animal Care and Use Committee of the Shanghai Medical College of Fudan University.

### 2.4 CRISPR–Cas9 genome editing in zebrafish embryos

By using reciprocal BLAST, two *PARD3* orthologs (*pard3aa* and *pard3ab*) were identified in the zebrafish genome. To identify single guide (sg) RNA sites targeting *pard3aa* and *pard3ab*, the E‐CRISP web tool (http://www.e‐crisp.org/E‐CRISP/) was used, and the designed oligos for expressing CRISPR–Cas9 guide RNAs (gRNAs) were synthesized by TSINGKE. The sequences of sgRNAs and primers for knockdown efficiency identification are listed in Table S1. The DNA templates for the in vitro transcription reactions were generated by PCR amplification, and the sgRNA was transcribed in vitro from the T7 promoter using a ScriptMAX T7 Transcription Kit (Ambion/Thermo Fisher Scientific).

A 1 nl cocktail containing Cas9 protein (Thermo Fisher Scientific) with or without sgRNAs was injected into one‐cell stage embryos at the following concentrations: 100 pg sgRNAs (g‐*pard3aa*: g*‐pard3ab* = 1:1) + 250 pg Cas9 per embryo. To assess the knockdown efficiency at the targeted genomic locus in F0 larvae, embryos were collected at 3 days post‐fertilization (dpf). Genomic DNA was extracted, DNA fragments containing sgRNA target sites were amplified by PCR, and PCR products were sequenced to detect knockdown efficiency.

### 2.5 Variant mRNA synthesis and embryo microinjection

The pTA2 vector (TOYOBO) containing *PARD3* truncated variant cDNAs was linearized by the NotI FD enzyme (Thermo Fisher Scientific) and purified using a DNA purification kit (Axygen). Variant mRNA was further synthesized by in vitro transcription using the mMESSAGE mMACHINE™ T7 ULTRA Transcription Kit (Thermo Fisher Scientific) with the linearized plasmid as the template. The mRNA was further purified with LiCl and then quantified with a Nanodrop spectrophotometer (Invitrogen). mRNAs were subpacked and stored at −80°C. For microinjection, 1 nl of the injection mix was delivered directly into the one‐cell staged embryos at a final concentration of 200 ng/μl.

### 2.6 Alcian blue staining

Zebrafish larvae were fixed in 4% PFA for 2 h at room temperature and then washed with PBS 3 times, followed by conventional gradient ethanol dehydration from 50% to 70%, including 50% twice and 70% once. Then, the larvae were stained overnight in staining liquid (0.02% Alcian blue, 0.005% alizarin red S and 50 mM MgCl_2_ in 70% ethanol) at room temperature. Larvae were washed 3 times with PBS and balanced with 1.5% H_2_O_2_ for 30 min at room temperature. Finally, the larvae were transferred to glycerin for dehydration and imaged using Leica imaging software.

### 2.7 Antibodies

A mouse monoclonal antibody against Flag (catalog number M20008S) was obtained from Abmart. The rabbit polyclonal antibody against PARD3 (catalog number 11085‐1‐AP) was purchased from Proteintech. Rabbit antibody against β‐catenin (catalog number 9562S) was obtained from Cell Signaling Technology. Peroxidase‐conjugated AffiniPure goat anti‐rabbit IgG (H+L), peroxidase‐conjugated AffiniPure goat anti‐mouse IgG (H+L), Alexa Fluor 488/594‐labelled goat anti‐rabbit IgG (H+L) and Alexa Fluor 488/594‐labelled goat anti‐mouse IgG (H+L) were purchased from Jackson Immunoresearch Laboratories.

### 2.8 Plasmids

Full‐length PARD3 (GenBank accession number NM_019619.4) was generated using basic PCR and subcloned into the pCDNA‐Flag (with SBP, His_8_ tag) vector. The truncating variants were constructed by using PCR and then subcloned into pCDNA‐Flag (with SBP, His_8_ tag) or pTA2 vector. The DNA oligonucleotides are listed in Table S1.

### 2.9 Cell culture, transfection, lentivirus packaging and infection

HEK‐293T and MCF‐10A cells were purchased from the American Type Culture Collection. HEK‐293T cells were cultured in Dulbecco’s modified Eagle’s medium (DMEM) plus 10% foetal bovine serum, while MCF‐10A cells were maintained in a monolayer in Dulbecco’s modified Eagle’s medium (DMEM/F12) (Invitrogen, 21041025) supplemented with 5% horse serum (Invitrogen, 16050122), 5% foetal bovine serum, 1% penicillin/streptomycin (Invitrogen, 15140122), 0.5 μg/ml hydrocortisone (Sigma, H‐0888), 100 ng/ml cholera toxin (Sigma, C‐8052), 10 μg/ml insulin (Sigma, I‐1882) and 20 ng/ml recombinant human EGF (Peprotech, 100‐15) at 37°C supplied with 5% CO_2_. HEK‐293T cells were transfected using LipofectamineTM 2000 (Invitrogen) as described in the manufacturer’s protocol. For stable transfection, packaging of the lentiviral full‐length PARD3 and truncated PARD3 expression constructs into pseudoviral particles was performed in cells using the pPACKH1TM HIV Lentivector Packaging kit according to the manufacturer’s instructions (LV500A‐1, Systems Biosciences). After 48 h, the virus‐containing supernatants were collected, filtered (with 0.45 μm syringe filters) and used to infect the cells with 5 μg/ml polybrene (Sigma). Forty‐eight to 72 h after infection, the infected stable cells were selected by a specific antibiotic (InvivoGen, puromycin dihydrochloride, 2 μg/ml for HEK‐293T cells or 5 μg/ml for MCF‐10A cells).

### 2.10 Three‐dimensional (3D) cell culture

Matrigel was spread evenly on precooled glass slides in a 24‐well plate and placed in an incubator to allow the Matrigel to solidify for 20–30 min. The adherent cultured MCF‐10A cells were digested by trypsin and resuspended in assay medium (DMEM/F12: 50 ml, horse serum: 1 ml, hydrocortizone (1 mg/ml): 5 μl, insulin (10 mg/ml): 50 μl and pen/strep: 500 μl) at a final density of 5000 cells/ml. The cell mixture was added to the chamber with Matrigel‐coated slides and cultured for 10 days to polarize the growth‐arrested acinar structure. More details about the 3D cell culture of MCF‐10A cells were conducted as described previously.^30^

### 2.11 Immunofluorescence (IF)

The MCF‐10A acinar structures were washed with PBS 2 times, fixed with 4% paraformaldehyde for 30 min at room temperature, permeabilized with 0.2% Triton X‐100 and then incubated with the appropriate primary antibodies overnight at 4 °C. The proteins were then incubated with goat anti‐mouse (or rabbit) FITC‐conjugated secondary antibody for 1 h at room temperature, and cell nuclei were visualized by DAPI staining. The slides were mounted with fluorescence decay‐resistant medium (Beyotime) and were observed under a confocal laser‐scanning microscope (Leica).

### 2.12 Mass spectrometry

HEK‐293T cells were stably transfected with pCDH‐SBP‐HIS_8_‐PARD3‐c.1012dupG. The cells were lysed, and Western blotting was performed to detect the expression. The SBP‐His_8_‐tagged truncated PARD3 protein was then precipitated by two‐step affinity purification with streptavidin‐agarose and then with nickel resin. The precipitate containing the complex copurified with SBP‐His_8_‐PARD3‐c.1012dupG was digested with sequencing grade trypsin (Promega). The resulting samples of peptides were analysed by LC–ESI–MS/MS using an Eksigent 2D nanoLC coupled in‐line with an LTQ‐OrbiTrap mass spectrometer. More detailed steps were conducted as we described previously.^5^

### 2.13 Western blot and coimmunoprecipitation (co‐IP) assay

Cells were collected, washed twice with precooled PBS and lysed in NP‐40 protein lysis buffer for 30 min at 4°C. The protein concentration was measured using a bicinchoninic acid assay kit (Thermo Fisher Scientific). Immunoblotting was conducted as described previously.^31^

For immunoprecipitation, total cell lysates were incubated with the appropriate antibodies for 2 h and then rotated with protein A/G beads overnight at 4°C. The beads were subsequently washed three times using NP‐40 lysis buffer and then mixed with 1× SDS loading buffer and boiled for 10 min, and the supernatant was analysed by immunoblotting.

### 2.14 Statistical analysis

Data are expressed as the means ± SEM. Statistical analysis was determined by Student’s *t* test, with *p < 0.05, **p < 0.01, and ***p < 0.001 indicating a significant difference.

## 3 RESULTS

### 3.1 Clinical evaluation of cases in the pedigree with nonsyndromic cleft palate and WES

**FIGURE 1:**
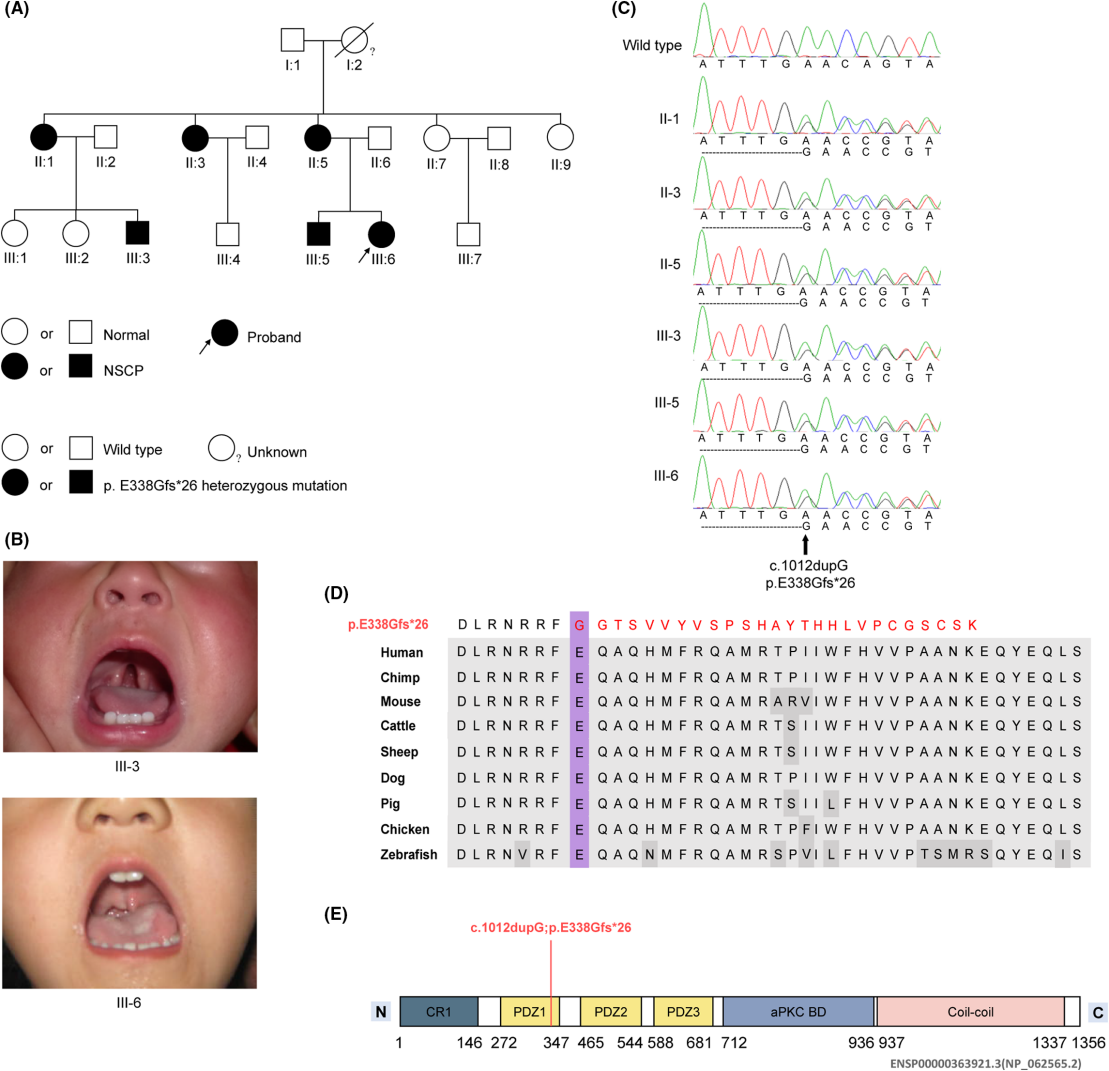
*PARD3* variant found in a pedigree with NSCP. (A) The pedigree with nonsyndromic cleft palate only. Unfilled and shaded shapes denote healthy and affected individuals, respectively. Squares represent males, and circles represent females. The arrow indicates the proband. (B) Clinical image of two affected individuals (III3 and III6) from the pedigree, which show the cleft of the soft palate. (C) The heterozygous single nucleotide insertion variant in *PARD3* was verified by Sanger sequencing. The variant was named according to GenBank: NM_019619.4, NP_062565.2. (D) Protein sequence alignment of PARD3 orthologs was performed by Multiple Sequence Alignment (MUSCLE). The p. E338Gfs*26 variant and the associated new residue sequence are shown above and indicated in red. (E) A schematic diagram of functional domains in the PARD3 (GenBank: NP_062565.2) protein. N= amino terminus; C= carboxy terminus

The family presented in this study originated from the Han population of China and was a consanguineous pedigree with three generations. Proband III:6 was first diagnosed with nonsyndromic cleft palate because the physical examination revealed a soft cleft palate. Pedigree analysis showed that the second generation had five individuals consisting of three affected individuals (II:1, II:3 and II:5) and two unaffected individuals (II:7 and II:9) (Figure 1A). Among the third generation, three were affected (III:3, III:5 and III:6), and the rest were healthy individuals. I:1 presented with a normal phenotype, while I:2 was deceased, and the phenotypic status could not be obtained. The affected individuals exhibited similar features hallmarked by a cleft of the soft palate (Figure 1B, Table 1), and the family tree showed an apparent autosomal‐dominant inheritance pattern.

**TABLE 1:** Variant and clinical summary of pedigree

Individual	Gender	Age (years)	Diagnosis	Genotype
I:1	M	62	Normal	wt/wt
II:1	F	35	Cleft of the soft palate	p.(E338Gfs*26)/wt
II:2	M	36	Normal	wt/wt
II:3	F	32	Cleft of the soft palate	p.(E338Gfs*26)/wt
II:4	M	33	Normal	wt/wt
II:5	F	28	Cleft of the hard and soft palate	p.(E338Gfs*26)/wt
II:6	M	31	Normal	wt/wt
II:7	F	24	Normal	wt/wt
II:8	M	27	Normal	wt/wt
II:9	F	21	Normal	wt/wt
III:1	F	10	Normal	wt/wt
III:2	F	6	Normal	wt/wt
III:3	M	1	Cleft of the soft palate	p.(E338Gfs*26)/wt
III:4	M	4	Normal	wt/wt
III:5	M	8	Cleft of the soft palate	p.(E338Gfs*26)/wt
III:6	F	5	Cleft of the soft palate	p.(E338Gfs*26)/wt
III:7	M	2	Normal	wt/wt

To identify the pathogenic gene in the family, we performed whole‐exome sequencing on samples from one normal control (II:9) and three affected individuals (II:5, III:3 and III:6). Variants were evaluated with two filtering criteria: (1) the minor allele frequency (MAF) was <0.001 in the public database (gnomAD database); (2) detected in the affected individual (II:5, III:3 and III:6) but absent in the unaffected family member (II:9). After filtering, fourteen candidate variants remained (Table S2). Sanger sequencing was carried out to verify the fourteen candidate variants and further detect their distribution in the family members, including the 6 affected and 11 unaffected family members whose DNA samples were available. Only the heterozygous single nucleotide insertion variant in *PARD3* (GenBank: NM_019619.4: c.1012dupG; NP_062565.2: p. E338Gfs*26) fully segregated with the disease in the 17 family members (Figure 1A,C and Table S2). Furthermore, the *PARD3* (c.1012dupG) variant had the highest CADD‐Phred score among the 14 variants and was not found in public databases (Table S2) or in our in‐house data from 300 unrelated controls. *DSP* (c.4751_4756delCGTCAG), *PIGR* (c.1699C>T) and *SPOCK3* (c.997C>T) were not found, and *CDC42BPB* (c.3926C>T) exhibited an ultralow allele frequency in the gnomAD database, while *GPR142* (c.316G>A), *LAMB3* (c.929C>T), *MAP3K5* (c.923T>G) and *TRPM5* (c.293C> G) were predicted to disrupt their protein functions, but they did not segregate with the disease in the 17 family members: being absent in some affected members and appeared in some normal ones (Table S2). Moreover, protein sequence alignment of p. E338Gfs*26 and PARD3 orthologs from human to zebrafish was performed, which showed that PARD3 was highly conserved across different species and that the p. E338Gfs*26 frameshift variant was located in the N‐terminal PDZ1 domain of PARD3 (Figure 1D,E).

### 3.2 *pard3aa* and *pard3ab* disruption resulted in aberrant ethmoid plate morphogenesis in zebrafish.

Palatogenesis is considered a good example of the conservation of genetic and morphogenetic events in vertebrate ontogeny. Zebrafish have been used in studying the genetic underpinnings of cleft palate, as the gene regulatory networks governing the differentiation of relevant tissues are relatively conserved between zebrafish and mammals.^32^, ^33^, ^34^, ^35^ Although the ethmoid plate in zebrafish is a cartilaginous structure and therefore not strictly homologous to the mammalian palate, it is often used as a proxy for the mammalian secondary palate because of its position at the roof of the oropharynx, the fact that it is formed through midline convergence, and that many orthologs of orofacial cleft genes are expressed there. In zebrafish, the *pard3aa* and *pard3ab* genes were identified as orthologs of PARD3 by using reciprocal BLAST (61% query cover, 68% identity; 87% query cover, 69% identity versus human protein, respectively).

**FIGURE 2:**
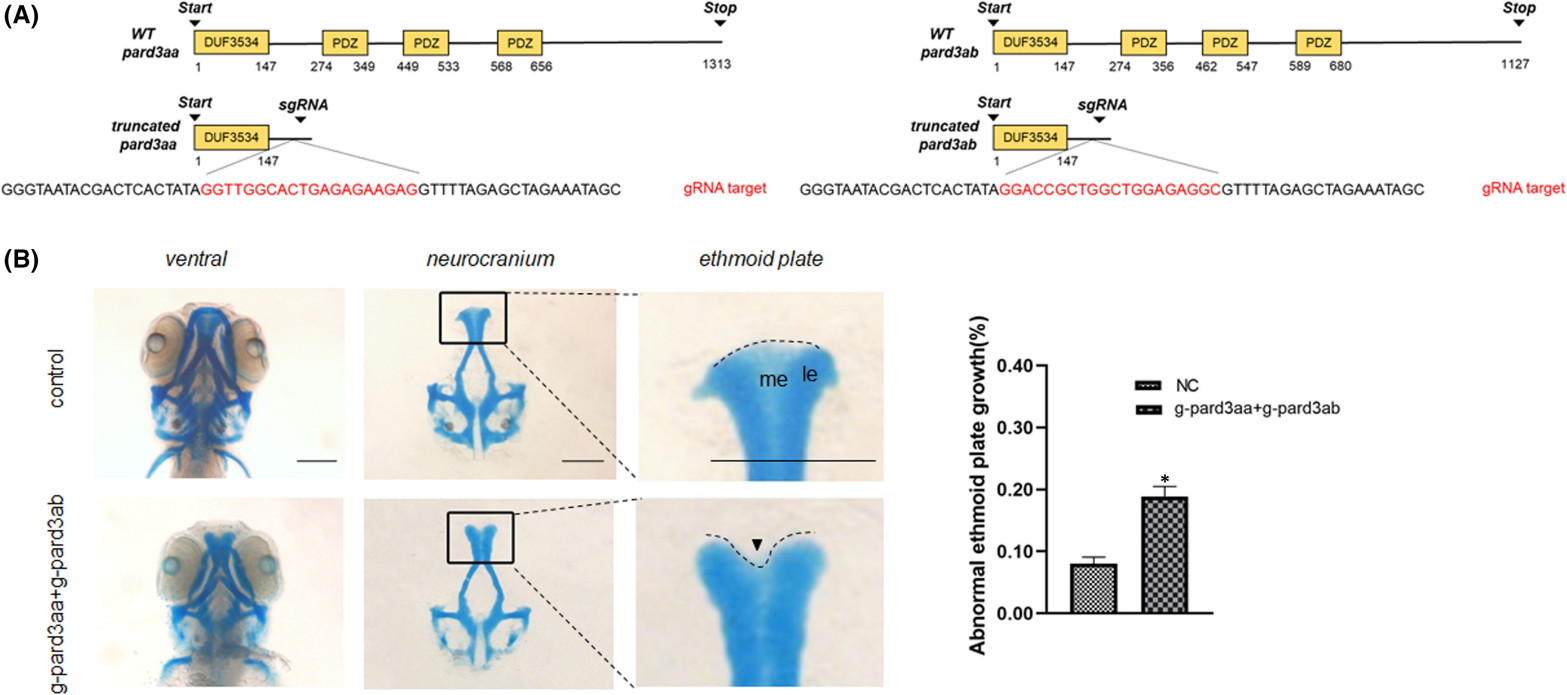
Zebrafish with *pard3aa* and *pard3ab* disruption displayed ethmoid plate patterning defects. (A) Schematic diagram of the binding and cleavage site of CRISPR/Cas9 in the coding sequence of the zebrafish PARD3 orthologs (*pard3aa*, *pard3ab*). Red = gRNA target. (B) Comparison of craniofacial structures of F0 CRISPR/Cas9‐mediated mutant zebrafish and Cas9 control zebrafish. The neurocranium was dissected at 3 dpf. Compared with the larvae in the control group, the CRISPR mutant larvae had a mild dysplasia phenotype, with subtle indentations at the upper edge of the ethmoid plate or hypoplastic at the median ethmoid (arrowheads). me = median ethmoid; le = lateral ethmoid. The statistical analysis of abnormal developmental palate was performed (bars indicate the means ± SEM. Student’s *t* test, *p < 0.05). Scale bar = 200 μm

To determine the function of orthologs of PARD3 in zebrafish ethmoid plate formation, sgRNAs were designed to target the N‐terminal coding sequence of *pard3aa* and *pard3ab* to knockdown their expression (Figure 2A). The sgRNAs were injected in the presence of Cas9 protein into embryos at the one‐cell stage, and obvious lethality was not observed after embryo injection. The efficacy of sgRNAs was confirmed by sequencing the PCR products across the target sequence (Figure S1) from genomic DNA of mixed zebrafish embryos 3 days after injection. Alcian blue staining was performed for analysis of the cartilaginous skeleton. Compared with the normal control group, in *pard3aa* and *pard3ab*‐disruption F0 mutants, middle ethmoid plate dysplasia was observed, specifically in the form of subtle indentations at the upper edge of the ethmoid plate or hypoplastic at the median ethmoid plate (Figure 2B).

### 3.3 Expression of the patient‐derived PARD3‐c.1012dupG variant induced aberrant ethmoid plate development in zebrafish.

**FIGURE 3:**
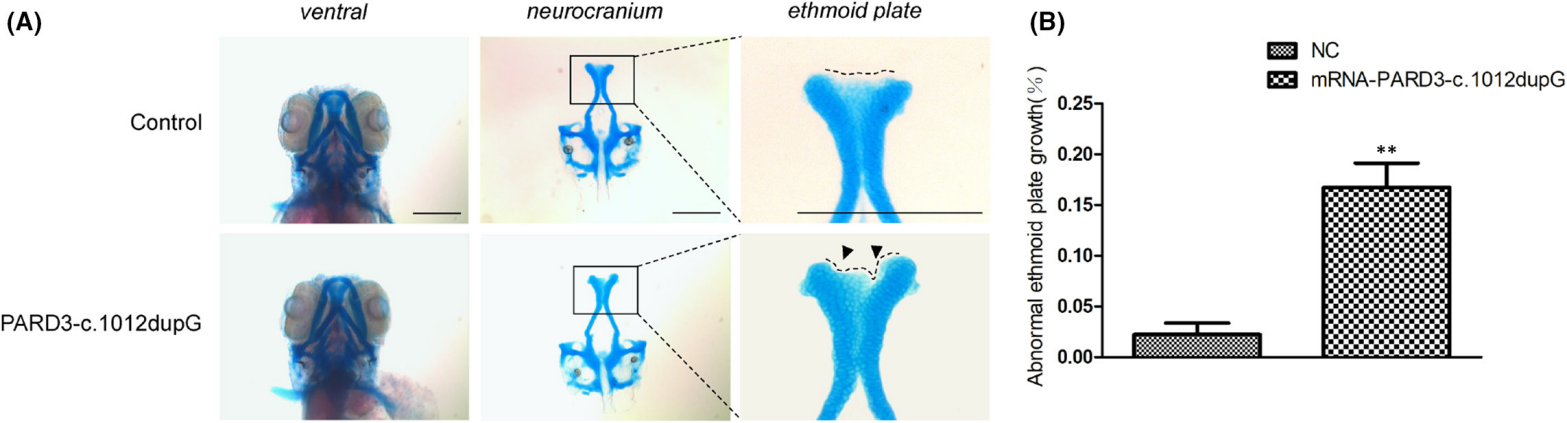
Expression of the patient‐derived truncated *PARD3* (c.1012dupG) variant induced hypoplastic ethmoid plate development in zebrafish. (A) Representative images of the control group and the larvae with patient‐derived truncating variant mRNA injection are depicted. Compared with the development of the ethmoid plate in control group larvae, the larvae injected with PARD3‐c.1012dupG mRNA showed ethmoid plate dysplasia, with the median ethmoid plate having a certain degree of absence and failing to form a smooth upper edge of the ethmoid plate. Scale bar = 200 μm. (B) Quantification of hypoplastic development of the ethmoid plate between the experimental groups and the control group (bars indicate the means ± SEM. Student’s *t* test was used to analyse the data. **p < 0.01)

PARD3‐c.1012dupG mRNA was injected into zebrafish embryos at the one‐cell stage, and the development of the ethmoid plate was observed at 3 dpf. Alcian blue staining revealed that the experimental group with expression of c.1012dupG mRNA exhibited ethmoid plate dysplasia with the median ethmoid plate having a certain degree of absence and failing to form a smooth upper edge of the ethmoid plate (Figure 3A,B), which indicated that the N‐terminal truncating PARD3 mutant may have gain‐of‐function effects on ethmoid plate development.

### 3.4 The truncated PARD3‐c.1012dupG mutant changed the localization of the wild‐type full‐length PARD3 protein.

**FIGURE 4:**
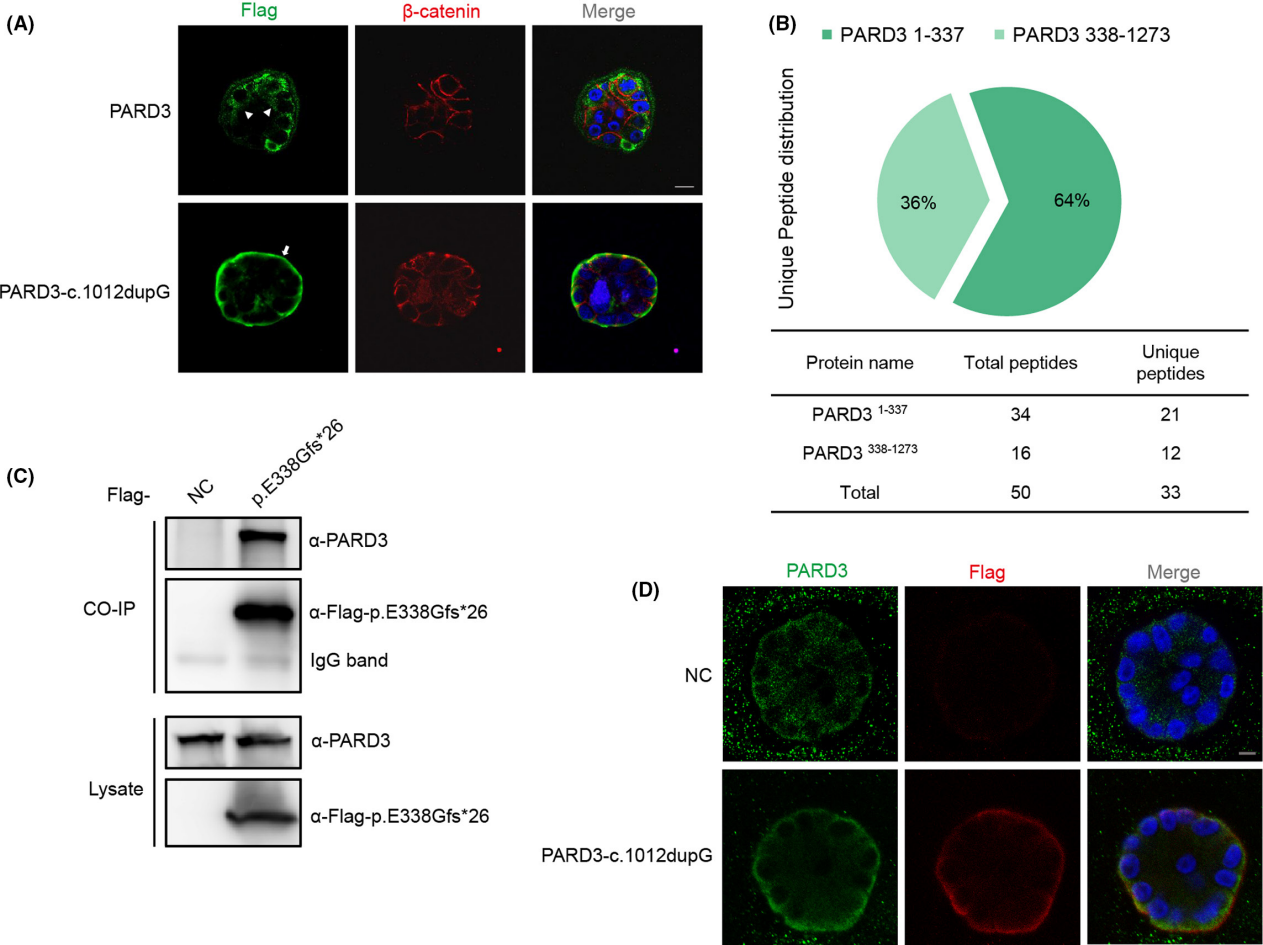
Truncated PARD3‐c.1012dupG variant changed the localization of the wild‐type full‐length PARD3 protein. (A) MCF‐10A cells stably expressing Flag‐tagged PARD3‐c.1012dupG or full‐length PARD3 were developed by lentiviral infection and puromycin selection and formed apical lumens after 10 days of 3D culture in Matrigel. The cysts were stained for Flag‐tagged PARD3 (wild‐type or mutant) (green) and the basolateral membrane marker β‐catenin (red). The truncated PARD3(c.1012dupG) was mainly localized to the basal compartment, while the full‐length PARD3 was mainly localized to the lateral and apical areas. The arrow points to the presence of truncated PARD3 at the basal region. Arrowheads point to the apical region. Scale bar = 10 μm. (B) Mass spectrometry analysis of PARD3‐c.1012dupG products identified endogenous full‐length PARD3 as the candidate interacting protein. Plasmids expressing SBP‐His_8_‐tagged PARD3‐c.1012dupG or empty vector were stably transfected into HEK‐293T cells, and the cells were harvested and lysed 72 h after selection with hygromycin B. Peptides derived from the trypsin digestion of mutant PARD3 pull down complex were analysed by LC–MS/MS. Herein, we used PARD3^338‐1273^ to refer to the C‐terminal signal of endogenous PARD3 bound by the PARD3‐c.1012dupG protein. The number of peptide hits for the C‐terminal signal of endogenous PARD3 (PARD3^338‐1273^) is shown as a pie chart and table. (C) Endogenous PARD3 interacted with Flag‐tagged PARD3‐p. E338Gfs*26. Flag‐tagged PARD3‐p. E338Gfs*26 was immunoprecipitated from the cell lysate of HEK‐293T cells stably expressing Flag‐tagged PARD3‐p. E338Gfs*26, and the coimmunoprecipitation product was analysed by anti‐PARD3 (C‐terminal immunogen) and anti‐Flag immunoblotting. (D) Substantial proportion of endogenous PARD3 colocalized with Flag‐tagged PARD3‐c.1012dupG mainly at the basement membrane in 3D‐cultured MCF‐10A cells. MCF‐10A cells stably expressing Flag‐tagged PARD3‐c.1012dupG or empty vector were grown in Matrigel, and endogenous PARD3 was analysed with immunofluorescent staining using C‐terminal PARD3 antibody (green). Mutant PARD3 was visualized by Flag antibody (red), and nuclei were stained with DAPI (blue). Scale bar = 7.5 μm

Epithelial cells have an apical surface facing a lumen or outside of the organism, while the basolateral surface faces other cells and extracellular matrix.^36^ PARD3 is asymmetrically localized in epithelial cells and plays an important role in the formation of the apical domain and cell–cell tight junction.^37^, ^38^ Epithelial MCF‐10A cells stably expressing wild‐type or truncated PARD3 were developed by lentiviral infection and formed spheroid structures in three‐dimensional Matrigel culture. In the MCF‐10A spheroid structures, wild‐type PARD3 was mainly detected at the tight junction site and apical region, while the truncated PARD3 (c.1012dupG) largely accumulated at the basal membrane (Figure 4A). To further explore the molecular mechanism of the patient‐derived truncated PARD3, HEK‐293T cells stably expressing the SBP‐His_8_‐tagged PARD3‐c.1012dupG protein were established, and the fused truncated protein was purified by SBP‐His_8_ tandem affinity purification (TAP). The complex copurified with PARD3‐c.1012dupG was digested with trypsin, followed by LC/MS mass spectrometry analysis. The mass spectrometry results revealed that, in addition to the peptides corresponding to truncated PARD3, many peptides located in the C‐terminus of PARD3 were also identified, which indicated that the PARD3‐c.1012dupG truncating protein (p. E338Gfs*26) bound the endogenous full‐length PARD3 (Figure 4B). To confirm the interaction between N‐terminal truncated PARD3 and full‐length endogenous PARD3, an anti‐Flag immunoprecipitation assay was performed in HEK‐293T cells stably expressing Flag‐tagged PARD3‐c.1012dupG, and endogenous PARD3 was analysed with a C‐terminal antibody that could not detect the PARD3‐c.1012dupG protein. The results revealed that the p. E338Gfs*26 truncating protein interacted with endogenous full‐length PARD3 (Figure 4C). Immunofluorescence analysis showed that a substantial proportion of endogenous PARD3 colocalized with Flag‐tagged PARD3‐c.1012dupG mainly at the basement membrane in 3D‐cultured MCF‐10A cells (Figure 4D).

### 3.5 A novel nonsense variant of *PARD3* was identified in sporadic cases with NSCP

We also performed a variant screening for the *PARD3* coding region in 57 NSCP sporadic cases. Among these cases, we identified a total of 7 variants, including one nonsense variant (NM_019619.3: c.397C>T) and six missense variants (NM_019619.3: c.718G>A, c.1723G>A, c.2201C>T, c.2402G>A, c.2620C>T and c.3205G>C) (Table 2). All of these variants were present as heterozygotes. Among these variants, two variants (NM_019619.3: c.397C>T, c.2201C>T) were not included in gnomAD data set, while the rest had an allele frequency of <0.01. Additionally, several bioinformatic tools, including Polyphen 2, SIFT and CADD, were used to predict protein function (Table 2). Also, c.718G>A, c.2201C>T, c.1723G>A and c.3205G>C were predicted to damage their protein functions by the Polyphen 2 or SIFT analysis. Furthermore, according to the American College of Medical Genetics (ACMG) guidelines, all the missense variants can be classified as VUS (Table S3).^39^, ^40^, ^41^ Among these variants found in the 57 sporadic NSCP cases, we detected the c.397C>T [p. R133*] nonsense variant, located within the N‐terminus of *PARD3*, which resulted in severe deletion and produced the truncated protein as the PARD3‐c.1012dupG variant in the pedigree with NSCP.

**TABLE 2:** Variants of *PARD3* in sporadic cases with NSCP

DNA change^a^	Protein change^b^	Genomic position^c^	Zygosity	CADD PHRED	Exon	Domain	Mutation type	gnomAD (allele frequency)	Polyphen2	SIFT	Category
c.397C>T	p.R133*	chr10:34516985	het	39	3	CR1	Nonsense	\	\	\	SNV
c.718G>A^d^	p.E240K	chr10:34401914	het	23.4	6	Before PDZ1	Missense	0.00005257	PD 0.573	Deleterious 0.00	SNV
c.2201C>T^d^	p.A734V	chr10:34347982	het	22.7	15	aPKC BD	Missense	\	PD 0.94	Tolerated 0.37	SNV
c.1723G>A	p.D575N	chr10:34360244	het	24.2	13	Before PDZ3	Missense	0.0001709	Benign 0.036	Deleterious 0.03	SNV
c.2620C>T	p.P874S	chr10:34331339	het	22.2	19	aPKC BD	Missense	0.00005259	Benign 0.040	Tolerated 0.40	SNV
c.2402G>A^e^	p.S801N	chr10:34341642	het	13.98	16	aPKC BD	Missense	0.003017	Benign 0.000	Tolerated 0.46	SNV
c.3205G>C	p.E1069Q	chr10:34269880	het	25.6	22	Coil–coil	Missense	0.0001512	PD 0.998	Deleterious 0.00	SNV

Abbreviations: CADD, combined annotation dependent depletion; PD, probably damaging; SIFT, sorting intolerant from tolerant.

^a^ Position on *PARD3* cDNA variant 1 (v1) RefSeq NM_019619.3.

^b^ Position on *PARD3* protein product RefSeq NP_062565.2.

^c^ Position according to the Homo sapiens GRCh38.p12 [GCF_000001405.38] chromosomes.

^d^ Variants were detected in one patient.

^e^ Variant was detected in two unrelated patients.

**FIGURE 5:**
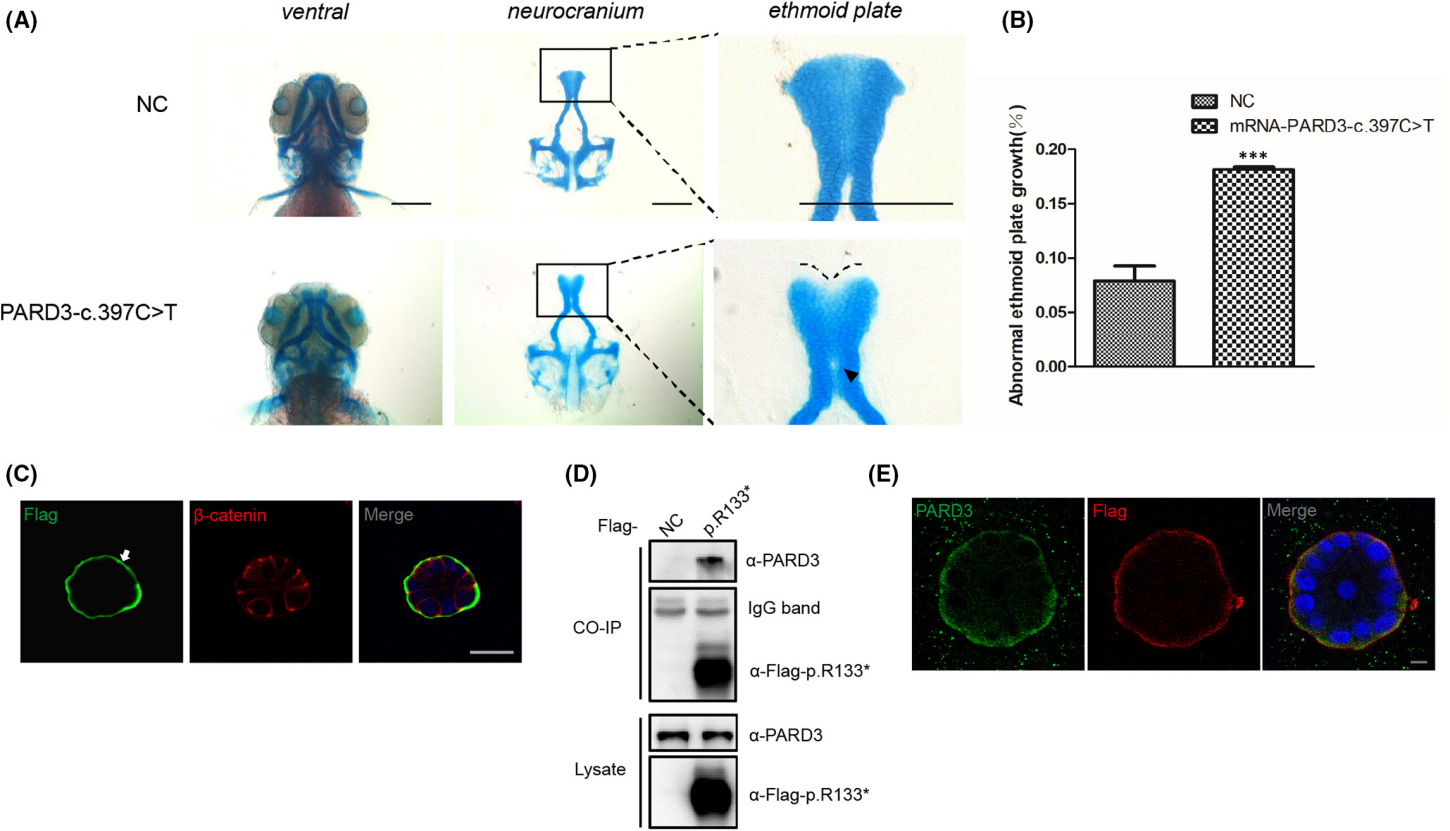
Functional validation of the N‐terminal truncating variant c.397C>T identified in a sporadic case with NSCP. (A) Representative images of the control group and zebrafish larvae injected with PARD3‐c.397C>T variant mRNA are depicted. Scale bar = 200 μm. (B) Statistical analysis for (A). Bars indicate the means ± SEM. Student’s *t* test was used to analyse the data. ***p < 0.001. (C) MCF‐10A cells stably expressing PARD3‐c.397C>T were polarized and formed apical lumens after 10 days of 3D culture in Matrigel. The cysts were stained for Flag‐tagged PARD3‐c.397C>T (green) and the basolateral membrane marker β‐catenin (red). PARD3‐c.397C>T localized to the basal compartment rather than the apical region. The arrowhead points to the presence of truncated PARD3 at the basal region. The asterisk indicates the apical area. Scale bar = 25 μm. (D) Endogenous PARD3 interacted with Flag‐tagged PARD3‐p.R133*. Flag‐tagged PARD3‐p. R133* was immunoprecipitated from cell lysates of HEK‐293T cells stably expressing Flag‐tagged PARD3‐p. R133*, and the coimmunoprecipitation product was analysed by anti‐PARD3 (C‐terminal immunogen) and anti‐Flag immunoblotting. (E) 3D culture of MCF‐10A cells was performed, and MCF‐10A cells stably expressing Flag‐tagged PARD3‐c. 397C>T showed that a substantial proportion of endogenous PARD3 colocalized with PARD3‐c.397C>T, which was mainly at the basement membrane. Endogenous PARD3 was analysed with immunofluorescent staining using a C‐terminal PARD3 antibody (green), PARD3‐c.397C>T was visualized with a Flag antibody (red), and nuclei were stained with DAPI (blue). Scale bar = 7.5 μm

Similar to the c.1012dupG variant, the nonsense variant c.397C>T resulted in an N‐terminal truncated PARD3 protein [p. R133*], so we further investigated its function and mechanisms. Human PARD3‐c.397C>T mRNA was injected into one‐cell stage zebrafish embryos, and ethmoid plate development was evaluated. Compared with the control group, ethmoid plate dysplasia with the median ethmoid plate showing a certain degree of absence and failure to form a smooth edge was observed in embryos with PARD3‐c.397C>T mRNA expression (Figure 5A,B), which was similar to PARD3‐c.1012dupG mRNA injection. In the 3D‐cultured MCF‐10A cells, the c.397C>T [p. R133*] variant also displayed mislocalization from the apical area to the basement membrane (Figure 5C). Similarly, immunoprecipitation assays and immunofluorescence analysis indicated that p. R133* bound endogenous full‐length PARD3 (Figure 5D), and the localization of endogenous full‐length PARD3 changed mainly near the basement membrane in 3D‐cultured MCF‐10A cells (Figure 5E).

## 4 DISCUSSION

Orofacial cleft is one of the most common birth defects, with an incidence of 1 in 700 people worldwide and is classified as syndromic orofacial cleft and nonsyndromic orofacial cleft.^1^ On the basis of anatomical, genetic and embryological findings, nonsyndromic orofacial clefts are commonly subdivided into nonsyndromic cleft lip with or without cleft (NSCL/P) and nonsyndromic cleft palate only (NSCP).^42^ Previous studies have implicated more than 26 risk chromosomal loci and candidate genes associated with nonsyndromic cleft lip with or without palate, with a few syndromic CL/P genes contributing to NSCL/P.^7^, ^8^, ^9^, ^43^, ^44^, ^45^ However, only limited research has been conducted on the genetic factors underlying NSCP.^10^, ^11^, ^12^, ^46^, ^47^, ^48^ In this study, the novel N‐terminal *PARD3* variant p. E338Gfs*26 cosegregated with cleft palate in the Chinese family with NSCP, and this variant was absent in three hundred unrelated controls. In the pedigree, the *PARD3*‐related NSCP was inherited in an autosomal‐dominant pattern.

Previous studies have indicated that palate development provides an excellent example of conservation of genetic programmes morphogenesis events across vertebrate ontogeny.^49^, ^50^, ^51^, ^52^, ^53^ Considering the high conservation of PARD3 across species, the zebrafish orthologs of *PARD3* were knocked down by CRISPR–Cas9/sgRNA targeting the N‐terminal coding sequence, to investigate its function in palatogenesis. Ethmoid plate dysplasia was observed in *pard3aa‐* and *pard3ab‐*disrupted zebrafish larvae, which implicated *pard3aa* and *pard3ab* in the development of the ethmoid plate.

PARD3, a member of the PARD family that was first identified in *C. elegans*, is a multimodular scaffold protein that plays key roles in cell tight junction and cell polarization processes.^54^, ^55^ To assess the impact of the variant on palatogenesis in zebrafish, PARD3‐c.1012dupG mRNA was injected into fertilized embryos at the one‐cell stage. We found that this led to ethmoid plate dysplasia, with the median ethmoid plate having a certain degree of absence and failing to form a smooth upper edge of the ethmoid plate contributing to the cleft phenotype, which indicated that the N‐terminal truncating variant of PARD3 may contribute to abnormal palatal development. PARD3 contains several evolutionarily conserved regions.^56^ It was previously reported that the N‐terminal domain CR1 is crucial for the apical localization and dimerization of PARD3.^55^ The N‐terminal domain‐mediated self‐association of the PARD3‐containing complex is also crucial for the formation of functional tight junctions.^57^ Our results revealed that PARD3‐p. E338Gfs*26 bound wild‐type full‐length PARD3 protein. Furthermore, the epithelial cell line MCF‐10A is a classic in vitro model for investigating apical–basal cellular polarity through the formation of a polarized growth‐arrested acini‐like spheroid, which has an apical surface facing the lumen and a basolateral surface facing the extracellular matrix.^30^, ^58^ In the polarized spheroid of MCF‐10A cells, PARD3‐p.E338Gfs*26 protein mainly localized near the basement membrane, while the localization of apical polarity and cell–cell junctions of wild‐type PARD3 was observed as previously described.^59^ Immunofluorescence analysis revealed that the localization of endogenous PARD3 was altered to the basal membrane in the 3D‐cultured epithelial cell model by colocalization with PARD3‐p. E338Gfs*26, which suggested that the N‐terminal truncating variant might have dominant‐negative effects on wild‐type PARD3. pLI has been broadly used in human genetics to help identify genes in which a single disrupting mutation is likely of clinical significance, as indicative of haploinsufficiency and dosage sensitivity.^60^, ^61^ The analysis showed that PARD3 was predicted to be highly intolerant to LOF mutations (probability of LOF intolerance, pLI = 0.94 in ExAC). Furthermore, besides dominant‐negative effects, frameshift and stop‐gain variants are also assumed to have haploinsufficiency or loss‐of‐function effects through degradation of the transcript by Nonsense‐Mediated Decay (NMD).^62^, ^63^, ^64^ We searched the Mouse Genome Informatics (MGI), the international database resource for the laboratory mouse, and found some information about the heterozygous deletion of *Pard3* in mice (MGI: 5756446), which showed that male and female *Pard3* ^+/‐^ mice displayed a weak phenotype: abnormal optic disc morphology, but no phenotypes of cleft palate were mentioned. Considering the above information and our findings that truncating PARD3 disrupts the localization of the wild‐type protein, we speculated that *PARD3* truncating variants had combined effects of haploinsufficiency and dominant‐negative in cleft palate.

Seven variants in the coding region of the *PARD3* gene were identified in 57 sporadic cases of NSCP. Except for one of the loci (c.2402G>A), the remaining observed allele frequencies in population database were <0.001 (gnomAD database), which indicated that these *PARD3* variants were rare in the normal population but could be found at a higher frequency in NSCP patients. Additionally, we noticed that the c.397C>T, c.2201C>T, c.2620C>T, c.2402G>A and c.3205G>C variants were all located in recorded conserved functional domains, while the c.718G>A and c.1723G>A variants were located near the PDZ domains. Based on these findings, several protein function prediction software programs (Polyphen 2, SIFT and CADD) were also used to assess the impact of variants on protein function and provide computational evidence to support the potential deleterious effect on the gene product. Except for the c.2402G>A variant, the remaining variants were predicted to disrupt the function of the encoded gene product according to the in silico prediction. Based on the evidence we have so far, and in the absence of additional supportive genetic or functional experimental evidence, these missense variants were classified as variants of uncertain significance (VUS) by the ACMG criteria. We anticipated that the inclusion of the *PARD3* locus in clinical testing would identify larger cohorts of pathogenic *PARD3* variants, and these cohorts would enable genotype–phenotype correlation studies. On the other hand, it is worth noting that among the 7 identified variants, c.397C>T also resulted in severe deletion and produced an N‐terminal truncating PARD3 protein. Our subsequent research revealed that expression of the c.397C>T variant also led to ethmoid plate dysplasia in zebrafish and displayed dominant‐negative effects on wild‐type PARD3 in the apical–basolateral polarization model of MCF‐10A cells.

In summary, the frameshift variant c.1012dupG in *PARD3* was identified as the candidate cause in the pedigree with NSCP, and the nonsense variant c.397C>T was also detected in sporadic NSCP cases, both of which produced N‐terminal truncated protein and displayed a dominant‐negative effect. Furthermore, 6 missense variants in *PARD3* found in our cohort of sporadic 57 NSCP cases suggests that missense variants may also contribute to cleft palate risk, although further investigation of the functional impact and role of such missense variants is warranted.

## AUTHOR CONTRIBUTIONS

**Jin Zhang:** Conceptualization (lead); funding acquisition (equal); methodology (lead); project administration (lead); writing – review and editing (equal). **Renjie Cui:** Conceptualization (equal); formal analysis (lead); methodology (lead); visualization (equal); writing – original draft (equal). **Dingli Chen:** Resources (equal); writing – original draft (equal). **Na Li:** Investigation (equal); methodology (equal). **Ming Cai:** Funding acquisition (equal); project administration (equal); resources (equal); writing – review and editing (equal). **Teng Wan:** Formal analysis (equal); resources (equal). **Xueqiang Zhang:** Investigation (equal); supervision (equal). **Meiqin Zhang:** Methodology (equal). **sichen du:** Data curation (equal). **huayuan ou:** Formal analysis (equal); methodology (equal). **Jianjun Jiao:** Software (equal); supervision (equal). **Nan Jiang:** Project administration (equal); supervision (equal). **Shuangxia Zhao:** Supervision (equal); validation (equal). **Xuedong Song:** Resources (equal); validation (equal). **Huaidong Song:** Methodology (equal); project administration (equal). **duan ma:** Conceptualization (equal); funding acquisition (lead); resources (equal); supervision (equal). **Shouxia Li:** Conceptualization (equal); project administration (equal); resources (equal).

## CONFLICT OF INTEREST

The authors declare that they have no conflicts of interest.

## Supporting information


Figure S1

Table S1

Table S2

Table S3

## Data Availability

The data that support the findings of this study are available from the corresponding author upon reasonable request.
