# Supplementary figures and images for: PARD3 gene variation as candidate cause of nonsyndromic cleft palate only
Source: J Cell Mol Med. 2022 Jul 4;26(15):4292–304. doi: 10.1111/jcmm.17452 (PMC9344820; doi:10.1111/jcmm.17452)

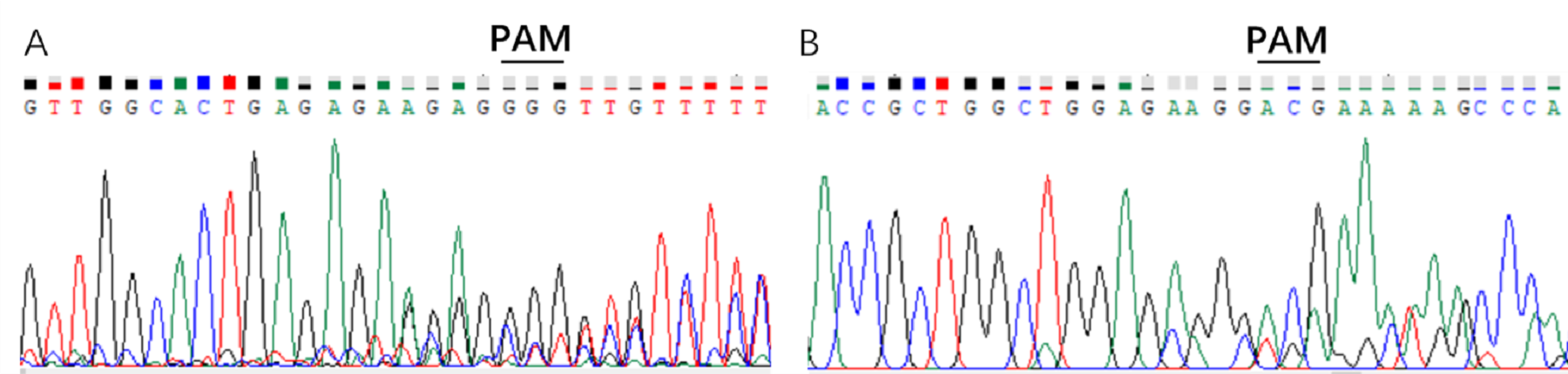

Supplement: Supplementary file 1 — Figure S1 [file JCMM-26-4292-s002.png]
