# Supplementary material for: PARD3 gene variation as candidate cause of nonsyndromic cleft palate only
Source: J Cell Mol Med. 2022 Jul 4;26(15):4292–304. doi: 10.1111/jcmm.17452 (PMC9344820; doi:10.1111/jcmm.17452)
Supplement: Supplementary file 2 — Table S1 [file JCMM-26-4292-s004.docx]

| **Supplemental Table 1 DNA oligonucleotides used in the present study (written 5’→3’)** | | |
| --- | --- | --- |
| Oligonucleotides | Foward | Reverse |
| zebra-T7-pard3aa-gRNA | GGGTAATACGACTCACTATAGGTTGGCACTGAGAGAAGAGGTTTTAGAGCTAGAAATAGC | AAAAGCACCGACTCGGTGCCACTTTTTCAAGTTGATAACGGACTAGCCTTATTTTAACTTGCTATTTCTAGCTCTAAAAC |
| zebra-T7-pard3ab-gRNA | TAATACGACTCACTATAGGACCGCTGGCTGGAGAGGCGTTTTAGAGCTAGAAATAGC | AAAAGCACCGACTCGGTGCCACTTTTTCAAGTTGATAACGGACTAGCCTTATTTTAACTTGCTATTTCTAGCTCTAAAAC |
| g-pard3aa-seq | AAACGATATTACCGCACACCT | CCCGACTATAAGAAACGTTGCA |
| g-pard3ab-seq | AAGGGCGTCTGTAACCATGT | TCCATAACTCAGCTCACCCG |
| hPARD3 | CTCGTCGACCATGAAAGTGACCGTGTGCTTC | GTGGCGGCCGCAGATCTCCTACACCGCTTAAAGGCAC |
| hPARD3-1012 bri | GACCTTCGAAATAGAAGATTTGGAACAAGCACAACATATGTTTC | GAAACATATGTTGTGCTTGTTCCAAATCTTCTATTTCGAAGGTC |
| hPARD3-1012 | CTCGTCGACCATGAAAGTGACCGTGTGCTTC | GTGGCGGCCGCAGATCTTTATTTGCTGCAGGAACCACATG |
| hPARD3-397 | CTCGTCGACCATGAAAGTGACCGTGTGCTTC | GTGGCGGCCGCAGATCTTCAAAGGACTGAAGGTGTGACC |
