# Supplementary material for: PARD3 gene variation as candidate cause of nonsyndromic cleft palate only
Source: J Cell Mol Med. 2022 Jul 4;26(15):4292–304. doi: 10.1111/jcmm.17452 (PMC9344820; doi:10.1111/jcmm.17452)
Supplement: Supplementary file 4 — Table S3 [file JCMM-26-4292-s001.docx]

| **Supplemental Table 3. ACMG pathogenicity evidence of *PARD3* variants** | | |
| --- | --- | --- |
| **DNA Change** | **Genomic Position** | **ACMG Evidence** |
| c.1012dupG | chr10:34673060 | Pathogenic：PVS1+PS3_supporting+PM2_supporting+PP1 |
| c.397C>T | chr10:34516985 | Pathogenic：PVS1+PM1+PS3_supporting+PM2_supporting |
| c.718G>A | chr10:34401914 | VUS：PP3 |
| c.2201C>T | chr10:34347982 | VUS：PM1+PM2_supporting |
| c.1723G>A | chr10:34360244 | VUS |
| c.2620C>T | chr10:34331339 | VUS：PM1 |
| c.2402G>A | chr10:34341642 | VUS：PS4+PM1+BP4_moderate |
| c.3205G>C | chr10:34269880 | VUS：PM1+PP3 |
